# *Plasmodium falciparum* antigenic variation: relationships between widespread endothelial activation, parasite PfEMP1 expression and severe malaria

**DOI:** 10.1186/1471-2334-14-170

**Published:** 2014-03-28

**Authors:** Abdirahman I Abdi, Gregory Fegan, Michelle Muthui, Esther Kiragu, Jennifer N Musyoki, Michael Opiyo, Kevin Marsh, George M Warimwe, Peter C Bull

**Affiliations:** 1 KEMRI-Wellcome Trust Research Programme, P.O. Box 230–80108, Kilifi, Kenya; 2 Nuffield Department of Clinical Medicine, John Radcliffe Hospital, University of Oxford, Oxford OX3, UK; 3 The Jenner Institute, University of Oxford, ORCRB, Roosevelt Drive, Oxford OX3 9DU, UK; 4 Department of Biochemistry and Chemistry, Pwani University, P.O. Box 195, 80108 Kilifi, Kenya

**Keywords:** Group A-like, Angiopoietin-2, Rosetting, Sequestration, Impaired consciousness, Cerebral malaria, Respiratory distress

## Abstract

**Background:**

*Plasmodium falciparum* erythrocyte membrane protein 1(PfEMP1) is a family of variant surface antigens (VSA) that mediate the adhesion of parasite infected erythrocytes to capillary endothelial cells within host tissues. Opinion is divided over the role of PfEMP1 in the widespread endothelial activation associated with severe malaria. In a previous study we found evidence for differential associations between defined VSA subsets and specific syndromes of severe malaria: group A-like PfEMP1 expression and the “rosetting” phenotype were associated with impaired consciousness and respiratory distress, respectively. This study explores the involvement of widespread endothelial activation in these associations.

**Methods:**

We used plasma angiopoietin-2 as a marker of widespread endothelial activation. Using logistic regression analysis, we explored the relationships between plasma angiopoietin-2 levels, parasite VSA expression and the two syndromes of severe malaria, impaired consciousness and respiratory distress.

**Results:**

Plasma angiopoietin-2 was associated with both syndromes. The rosetting phenotype did not show an independent association with respiratory distress when adjusted for angiopoietin-2, consistent with a single pathogenic mechanism involving widespread endothelial activation. In contrast, group A-like *PfEMP1* expression and angiopoietin-2 maintained independent associations with impaired consciousness when adjusted for each other.

**Conclusion:**

The results are consistent with multiple pathogenic mechanisms leading to severe malaria and heterogeneity in the pathophysiology of impaired consciousness. The observed association between group A-like PfEMP1 and impaired consciousness does not appear to involve widespread endothelial activation.

## Background

Severe life threatening *P. falciparum* malaria is a major cause of mortality and morbidity in young children in sub-Saharan Africa. In endemic areas, severe malaria is most prevalent in children under the age of five years, before they acquire immunity to severe disease as a result of repeated exposure. Severe malaria manifests in children in three partly overlapping syndromes; impaired consciousness (IC), respiratory distress (RD), and severe malarial anemia (SMA) [1]. Of these, IC and RD were found to be the key indicators of life threatening malaria in a hospital setting in sub-Saharan Africa [1].

The pathophysiological process underlying each of these syndromes is still not understood. Disturbed microcirculation is thought to play a major role [2]. In cerebral malaria (CM) (severely impaired consciousness), sequestration of parasite infected erythrocytes (IE) in the microvasculature of the brain is thought to be important [3-6].

PfEMP1, a parasite encoded protein expressed on the surface of the IE, interacts with host receptors on the microvascular endothelia as well as unparasitized erythrocytes (a phenotype referred to as rosetting) leading to sequestration of the IE in organs. PfEMP1 is therefore thought to play a central role in parasite virulence. PfEMP1 is encoded by about 60 *var* genes per parasite genome and undergoes antigenic variation. Switches in the expression of the repertoire of *var* genes results in a high degree of plasticity in the antigenic and adhesive properties of the infecting parasite population.

Epidemiological studies have shown a subset of *var* genes preferentially expressed in young and non-immune children to be associated with severe malaria especially in children with IC [7-10]. This is consistent with 1) the hypothesis that some PfEMP1 variants have growth advantage in immunologically naïve children as the result of exhibiting a superior ability to sequester [11-18] and 2) the observed relationship between the density of sequestration in vital organs such as the brain and fatal malaria [4,5,18].

Beside sequestration of IE, severe malaria is characterised by systemic endothelial activation and widespread release of activation markers such as von Willebrand factor (vWF) [19], soluble ICAM-1(sICAM-1) [20] and angiopoietin-2 (ang-2) [21,22]. As sequestration occurs in the endothelial cells (ECs) of the microvasculature, it is believed that PfEMP1 mediated adhesion of parasitized red blood cells to the host microvasculature induces endothelial activation compromising the vascular integrity [23,24]. Recently, endothelial activation markers such as ang-2, soluble Tie-2 receptor, vWF have been shown to be associated with severe malaria [22,25]. Furthermore ang-2 is associated with retinopathy [25], a feature identified as a surrogate marker for cerebral sequestration [5,26] and a recent study found fibrin deposition in the brain to be associated with sequestration of IE [27]. If there is a connection between parasite *var* expression patterns and disease severity, through mechanisms involving sequestration and endothelial activation we would expect to observe a relationship between the expression of the *var* subset associated with severe malaria and markers of endothelial activation.

Previously, we showed through *var* expression profiling of 217 clinical *P. falciparum* isolates, that expression of a specific subset of “group A-like” PfEMP1 types is associated with severe malaria [8]. This was based on PCR amplification of a region within the DBLα domain of PfEMP1 and sequencing. Moreover, we showed that expression of these group A-like PfEMP1 types is associated primarily with the severe syndromes of IC, while the parasite rosette phenotype was associated primarily with RD [8,10]. In previous post-mortem studies of Thai adults [3,4] and African children [5], the severity of IC was shown to correlate with sequestration of IE by direct binding to the vascular endothelia [18]. The subgroup of *vars* expressed by the infecting parasites on the surface of IE may therefore determine the level of endothelial activation as suggested by [28].

In this study, we explored whether a relationship exists between widespread endothelial activation (represented by plasma ang-2 levels), parasite VSA expression and severe malaria. Specifically, we investigated whether widespread endothelial activation could provide a causal link between the expression of the group A-like *vars* and IC [10] on the one hand and rosette frequency and RD [10] on the other. To this end we measured ang-2 levels in the plasma of children from Kilifi, Kenya, presenting with either severe or non-severe malaria.

## Methods

### Ethics statement

Ethical approval for this study was obtained from Kenya Medical Research Institute (KEMRI) Ethical Review Committee (SSC 1131), and written, informed consent was obtained from parents/guardians of the study participants.

### Sample collection and clinical classification of patients

The method of sample collections was described in detail in [8,10]. Severe malaria cases include children with microscopically confirmed *P. falciparum* infection and admitted to the hospital ward with impaired consciousness (Blantyre coma score (bcs) <5 in patients aged ≥8 months or ≤3 in patients aged under 8 months) [29], respiratory distress (deep “Kussmaul” breathing) [30], severe malarial anemia (haemoglobin <5 g/dl) [1]. The severe form of impaired consciousness was defined as cerebral malaria (bcs ≤ 2).

### Sampling of DBLα-tag sequence and classification

The method for amplification of DBLα-tag from cDNA has been described in [31] and sequence classification for the dataset used in this study is described in [8,10]. Briefly, a blood sample was taken from all the children at admission. After removal of the white blood cells (WBC), an aliquot (~100 μl) of the erythrocyte portion was directly re-suspended in TRIzol and kept at −80 degrees till use. RNA was extracted from the frozen TRIzol and cDNA synthesized using random hexamers. Using degenerate primers a highly conserved region within the DBLα-domain of *vars* was PCR amplified, cloned into TOPO T-cloning vector, transformed into *E. coli*, and 100 colonies from each sample sequenced. The sequences were classified as described in [31,32]. Briefly they were classified based on the number of cysteine residues present (majority containing either two cysteine (cys2) or four cysteine (cys4) [31]. The sequences were also classified using network analysis according to whether they fall into groups that tend not to share polymorphic regions [33]. Group A-like *vars* belong to cys2 and block sharing group 1 [33]. Each subgroup was expressed as a percentage of the total sequences.

### Rosetting, IE surface antibody, and ang-2

Rosette frequency data was obtained as described previously [10]. IE surface antibodies data was obtained as described in [10]. Briefly we measured each participant’s IgG antibody levels (acquired as mean fluorescent intensity (MFI)) to the infected erythrocyte surface of eight ex vivo clinical isolates grown to the trophozoite stage using flow cytometry. The eight isolates were selected on the basis of their *var* expression (ranging from high to low cys2 expression). The median MFI value of this IE surface recognition by IgG against the eight isolates was calculated for each participant [10].

Ang-2 level in the acute plasma was determined by a commercial ELISA Kit from R & D (cat no; DANG20) using the manufacture’s protocol.

### PfHRP2 ELISA

Plasma PfHRP2 level was determined from the acute plasma using ELISA. Three plasma samples from patients with high *P. falciparum* parasitemia were used to determine both the dynamic range and the concentration of PfHRP2 using recombinant PfHRP2 protein obtained from MyBioSource (Cat no MBS232321). These plasma samples were then used to construct a standard curve. The ELISA assay was performed in duplicates in dilutions ranging from 1: 50 to 1:2000 and concentration (ng/ml) calculated from the linear range of the standard curve. Samples with OD readings greater than 20% standard deviation in the duplicate well was repeated or excluded from the analysis.

### Statistical analysis

Stata version 11 was used for all statistical analyses and P ≤0.05 was considered significant. Correlations between variables were evaluated using Spearman’s rank correlation coefficient or the 2-sample Wilcoxon rank-sum (Mann–Whitney) test. Linear regression was used when the outcome is a continuous variable and logistic regression when binary. All the regression analyses were adjusted for age. All independent variables that were significant in a univariate analysis were included in models with, either IC, or RD variable. Before use in a regression analysis, parasite density both peripheral and total (PfHRP2) and ang-2 were log-transformed. Rosette frequency and group A-like expression were arcsine-transformed as described in [8,10]. For all the logistic regression analysis where IC, RD, or cerebral malaria is the outcome, the syndrome was considered as a factor [10], i.e. cases are those positive for the syndrome (whether pure or mixed) and controls are those without the syndrome of interest (whether severe or non-severe).

We tested the normality of residuals in linear regression models by examining the interquartile range of the standardized residuals. None of the models had severe outliers, giving no sufficient evidence to reject normality at a 5% significance level.

We used the Hosmer-lemeshow goodness of fit test to examine the goodness of fit for all logistic regression models with all the models reported showing adequate fit (P > 0.05). We also used the likelihood ratio (LR) χ^2^ improvement test to assess the effect of adding subsequent explanatory variables to the fit of a simpler regression model.

## Results

### Patients characteristics

The samples used were from a published study [8] that involved 217 study subjects. Of these, 213 had sufficient plasma available to be included (see Additional file 1: Figure S1 for patient characteristics). Previously, the *var* expression profiles of the infecting parasites from these children were determined [10] together with a measure of the breadth of antibodies against antigens on the surface of the IE [8] present at the time of disease. Rosette frequency was determined for parasites from 130 of the samples [10].

We used ang-2 as a marker of widespread endothelial activation because unlike sICAM-1 [34] and vWF [35,36] that can be released from non-ECs or ang-1 which is entirely from non-endothelial source, ang-2 is only released from activated ECs [37,38]. Moreover, ang-2 is relatively stable under freeze-thaw cycling [39].

### Ang-2 levels are associated with the severe malaria syndromes; impaired consciousness and respiratory distress

**Figure 1:**
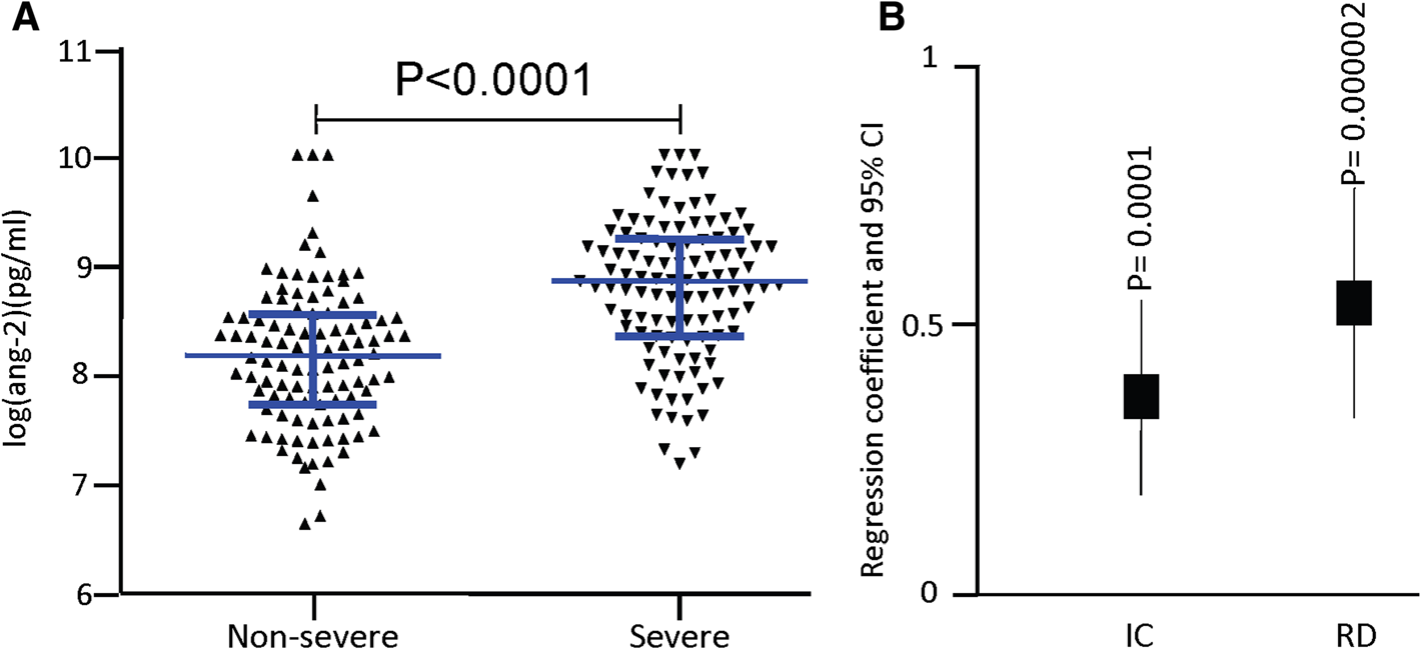
**Plasma ang-2 level and clinical malaria (N = 213). A)** Plasma ang-2 level and severe malaria. Shown in blue is the median and interquartile range, *P*-value determined by Mann–Whitney *U* test. **B)** Relationship between ang-2 plasma level and severe malaria syndromes: Plot of regression coefficient and 95% confidence interval obtained from an age-adjusted multi-variable regression model that predicted ang-2 using impaired consciousness and respiratory distress as explanatory variables (N = 213).

Consistent with previous studies [21,25], analysis of 213 children’s plasma showed that ang-2 levels were higher in children with severe malaria compared to those who were non-severe (Figure 1A: Mann–Whitney *U* test Z = 6.5, P < 0.0001, N = 213).

Since severe malaria in African children presents in three overlapping syndromes [1], these syndromes may be differentially associated with markers of endothelial activation. To explore this, we used ang-2 as the dependent variable in a multivariable linear regression model that considered host age, IC, and RD as explanatory variables (SMA was not included due to few samples with this clinical phenotype). Both RD and IC displayed significant and independent associations with ang-2 (Figure 1B) suggesting that endothelial activation may play a role in the pathophysiology of both syndromes.

### The association of IE surface antigen profiles with plasma angiopoietin-2

**Figure 2:**
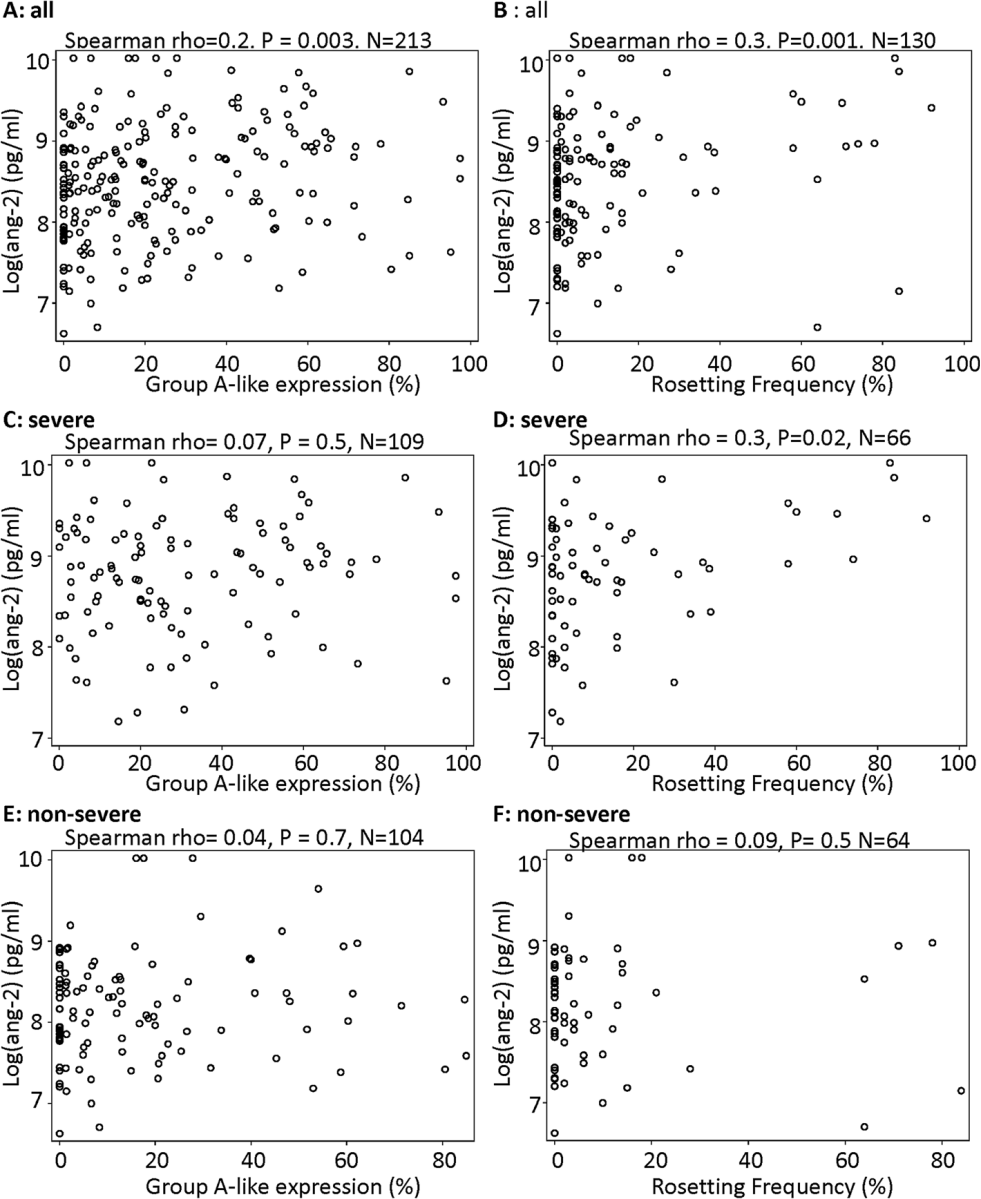
**The relationship between ang-2 and group A-like expression and the rosetting phenotype of the infecting parasite.** Scatter plots showing the relationship between **A)** ang-2 and group A-like expression, **B)** ang-2 and rosetting frequency for all the samples. **C & D** and **E & F** are repeats of **A** and **B** analysis within severe and non-severe cases respectively.

Given the potential link between cytoadherence and endothelial activation we explored two measures of variant surface antigen (VSA) expression: rosetting and group-A like *var*, in relation to ang-2 levels. A significant positive correlation was found between ang-2 and both group A-like *var* expression (Figure 2A) and rosetting (Figure 2B). To explore whether these relationships between the measures of VSA and ang-2 were due to direct parasite:host interactions, we tested whether the associations were maintained when we split the analysis into severe and non-severe cases (Figure 2C-F). Neither group A-like expression nor rosetting showed evidence for an association with ang-2 among non-severe cases (Figure 2E-F). This suggests that disease severity is a confounder, i.e. the observed associations between VSA expression and ang-2 are due to the fact that both are associated with disease severity rather than their direct associations with each other.

### Group A-like expression and ang-2 are independently associated with impaired consciousness

We explored further whether the previously observed relationship between the group A-like *var* subgroup expression and IC [10] may involve widespread endothelial activation. We did not consider rosetting here because we showed previously that it has no association with IC within this dataset [10].

**Figure 3:**
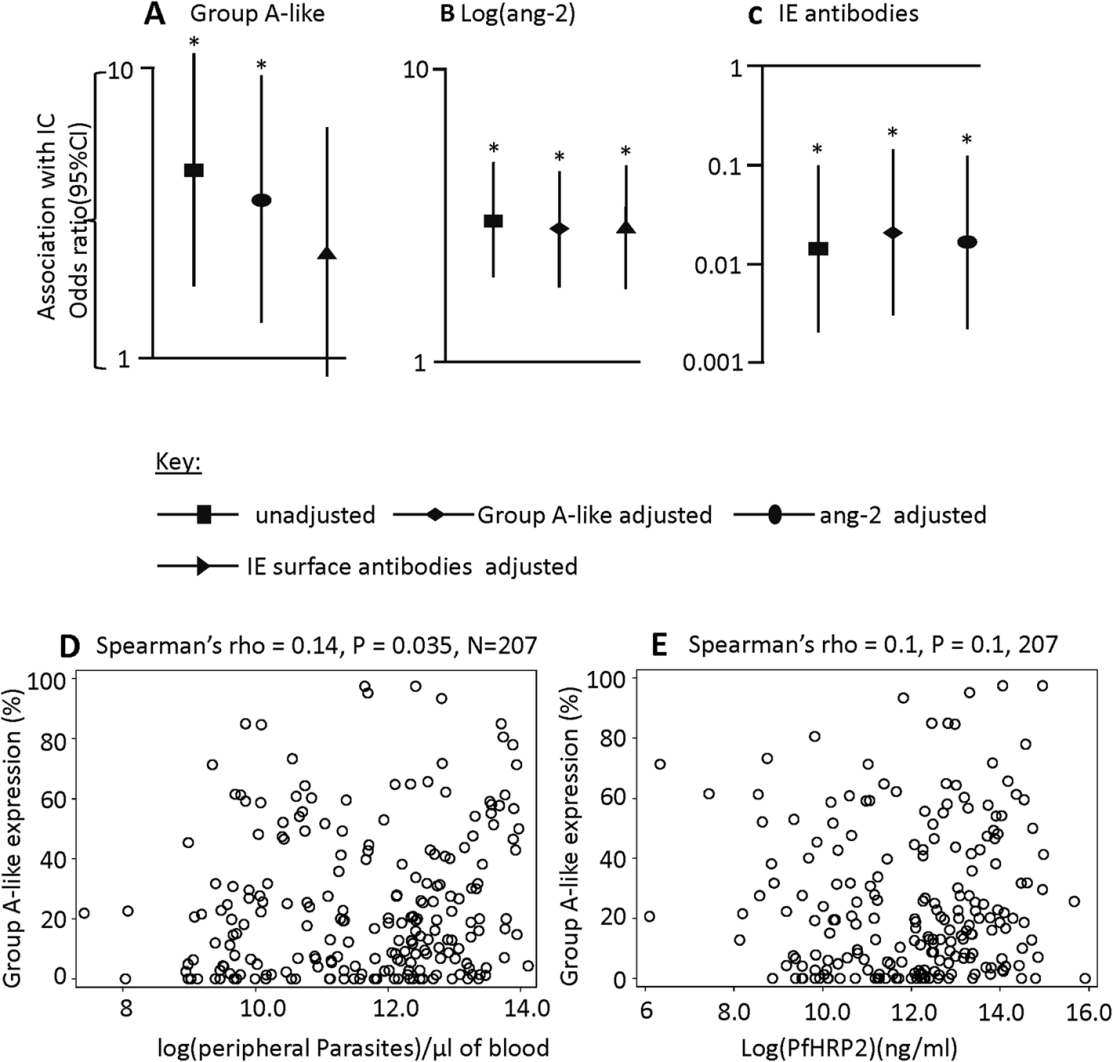
**Group A-like var expression and ang-2 have independent association with impaired consciousness.** A plot of odds ratio and 95% CI obtained from age-adjusted logistic regression models predicting impaired consciousness using; **A)** Group A-like expression before and after adjusting either for ang-2 or IE surface antibodies **B)** plasma ang-2, before and after adjusting for either group A-like expression or IE surface antibodies **C)** IE surface antibodies, before and after adjusting for either group A-like expression or ang-2. Asterisk indicate significance. Scatter plots showing the relationship between **D)** group A-like expression and peripheral parasite density, **E)** group A-like expression and PfHRP2.

We used age-adjusted logistic regression models predicting IC with group A-like *var* expression and ang-2 as independent variables. First, each of them was used as the only explanatory variable in an age-adjusted model and then in combination to adjust for one another. As shown in Figure 3A and Table 1, model 6A, despite a moderate reduction in the odds ratio (OR) when adjusted for ang-2, the group A-like *var* subgroup expression remained significantly associated with IC. Similarly, ang-2 remained significantly predictive of IC when adjusted for group A-like *var* expression (Figure 3B and Table 1, model 6A). A positive improvement to the fit of the model was observed when both variables were considered compared to either considered alone (likelihood ratio χ^2^ = 22.5, P < 0.001 and χ^2^ = 6.5, P < 0.01 respectively, compared to group A-like and ang-2 considered alone). These results suggest that the group A-like *var* expression and ang-2 are independently associated with IC.

**Table 1:** Age-adjusted logistic regression models predicting (A) impaired consciousness and (B) cerebral malaria (N = 213)

	**A: impaired consciousness**		**B: cerebral malaria**		
**Models**	**Explanatory variables**	**OR (95% CI)**	***P value***	***OR (95% CI)***	***P value***
1$	Group A-like	4.5(1.7,11.4)	0.002	2.97(1.14, 7.76)	0.026
2	ang-2	3.0(1.9, 4.7)	0.000002	2.52(1.57, 4.03)	0.00012
3$	Parasite density (peripheral)	1.37(1.12, 1.67)	0.002	1.46(1.16, 1.84)	0.001
4	PfHRP2	1.34(1.13, 1.60)	0.001	1.43(1.17, 1.74)	0.0004
5$	IE surface antibodies	0.96(0.94, 0.98)	0.00002	0.96(0.94, 0.99)	0.001
6	Group A-like	3.5(1.32, 9.44)	0.012‡	2.3(0.84,6.33)	0.1
	ang-2	2.82(1.78, 4.46)	0.000009‡	2.38(1.48, 3.82)	0.0003‡
7$	Group A-like	2.3(0.86, 6.3)	0.097	1.73(0.63, 4.77)	0.3
	IE surface antibodies	0.96(0.94, 0.98)	0.0001‡	0.97(0.95, 0.99)	0.003‡
8	ang-2	2.87(1.76, 4.66)	0.00002‡	2.35(1.43, 3.85)	0.001‡
	IE surface antibodies	0.96(0.94, 0.98)	0.00007‡	0.97(0.95, 0.99)	0.003‡
9$	Group A-like	4.15(1.6, 10.7)	0.003‡	2.65(0.99, 7.08)	0.05
	Parasite density (peripheral)	1.3(1.1, 1.65)	0.005‡	1.43(1.14, 1.81)	0.002‡
10	Group A-like	4.24(1.6, 11.21)	0.004‡	2.68( 0.98,7.37)	0.05
	PfHRP2	1.32(1.12, 1.57)	0.001‡	1.40(1.15, 1.71)	0.001‡
11	ang-2	2.7(1.7, 4.3)	0.00005‡	2.17(1.33, 3.53)	0.002‡
	Parasite density (peripheral)	1.21(0.97, 1.5)	0.09	1.32(1.04, 1.68)	0.023‡
12	ang-2	2.6(1.60, 4.24)	0.005‡	2.07(1.24, 3.44)	0.005‡
	PfHRP2	1.18(0.98, 1.41)	0.080	1.28(1.04,1.58)	0.019‡

The analysis in this table considers the outcomes IC and CM as factors, which means non-IC/CM severe cases are included in the control group and hence the analysis is conservative. The association of group A-like with impaired consciousness is independent of ang-2 (model 6). Similarly the IE surface antibodies and ang-2 show independent association with impaired consciousness (Model 8A, compare with model 7A). In Model 6B, the association of group A-like expression with CM is not statistically significant when ang-2 was considered. However, the odd ratios of both the unadjusted and ang-2 adjusted are similar in magnitude and direction. $ indicate models that have been published in [8,10] and repeated here for comparison. ‡ = variables that improved the fit of the model using LR χ^2^ improvement test. PfHRP2 data was obtained for 207 out of the 213 samples.

Next we examined the relationship between ang-2 levels and carriage at the time of disease of antibodies to the IE surface. Previously [8,10] we measured levels of these antibodies against 8 parasite isolates by flow cytometry and the median value of this IE surface recognition by IgG was calculated (henceforth called “IE surface antibodies”). IE surface antibodies showed a negative association with group A-like expression and IC [10]. We asked whether this negative association with IC might involve a causal network that includes ang-2. This would be expected if, for example, IE surface antibodies, by inhibiting cytoadherence mediated by group A-like PfEMP1 protect against endothelial activation and subsequently IC. We therefore tested the independence of IE antibodies, ang-2 and IC using logistic regression. As illustrated in Figure 3A and Table 1 model 7A, adjusting for IE surface antibodies reduced the estimated relationship between group A-like and IC, but showed no effect on the relationship between ang-2 and IC (Figure 3B and Table 1 model 8A). Similarly, adjusting for ang-2 did not alter the relationship between IE surface antibodies and IC (Figure 3C). Thus unlike group A-like *var* expression, the relationship between IE antibodies and IC appears to be independent of ang-2. Overall, a similar observation was made when CM (bcs ≤ 2) was considered as the outcome (Table 1 model 1B, 6B, 7B and 8B).

Overall, the independence between group A-like *var* expression and IE antibodies on the one hand and ang-2 on the other, in relation to their associations with IC suggest that distinct pathways may link group-A like *var* expression and widespread endothelial activation to IC.

Previous analysis of this dataset suggested that the association between group A-like *var* expression and IC is independent of peripheral parasite density [10]. We explored this further with another plasma marker, PfHRP2 (thought to be a marker of parasite burden within the host [40]). PfHRP2 has recently been found to have a strong relationship with retinopathy positive cerebral malaria [41]. To test the effect of PfHRP2 on the relationship between group A-like expression and IC, we measured plasma PfHRP2 and tested its relation with group A-like expression and ang-2 using Spearman’s rank correlation. Consistent with the observation made with the peripheral parasitemia (Figure 3D), group A-like var expression showed no evidence for an association with PfHRP2 (Figure 3E). Furthermore, both group A-like expression and PfHRP2 showed independent associations with IC in an age-adjusted logistic regression analysis (Table 1 model 10). In contrast PfHRP2 and ang-2 showed evidence for non-independence in their associations with IC (Table 1, model 12). These results suggest that group A-like expression and ang-2 have contrasting relationship with parasite load and the latter seem to be more important for ang-2 release.

### Associations between Rosette frequency, parasitemia, endothelial activation, and respiratory distress

**Figure 4:**
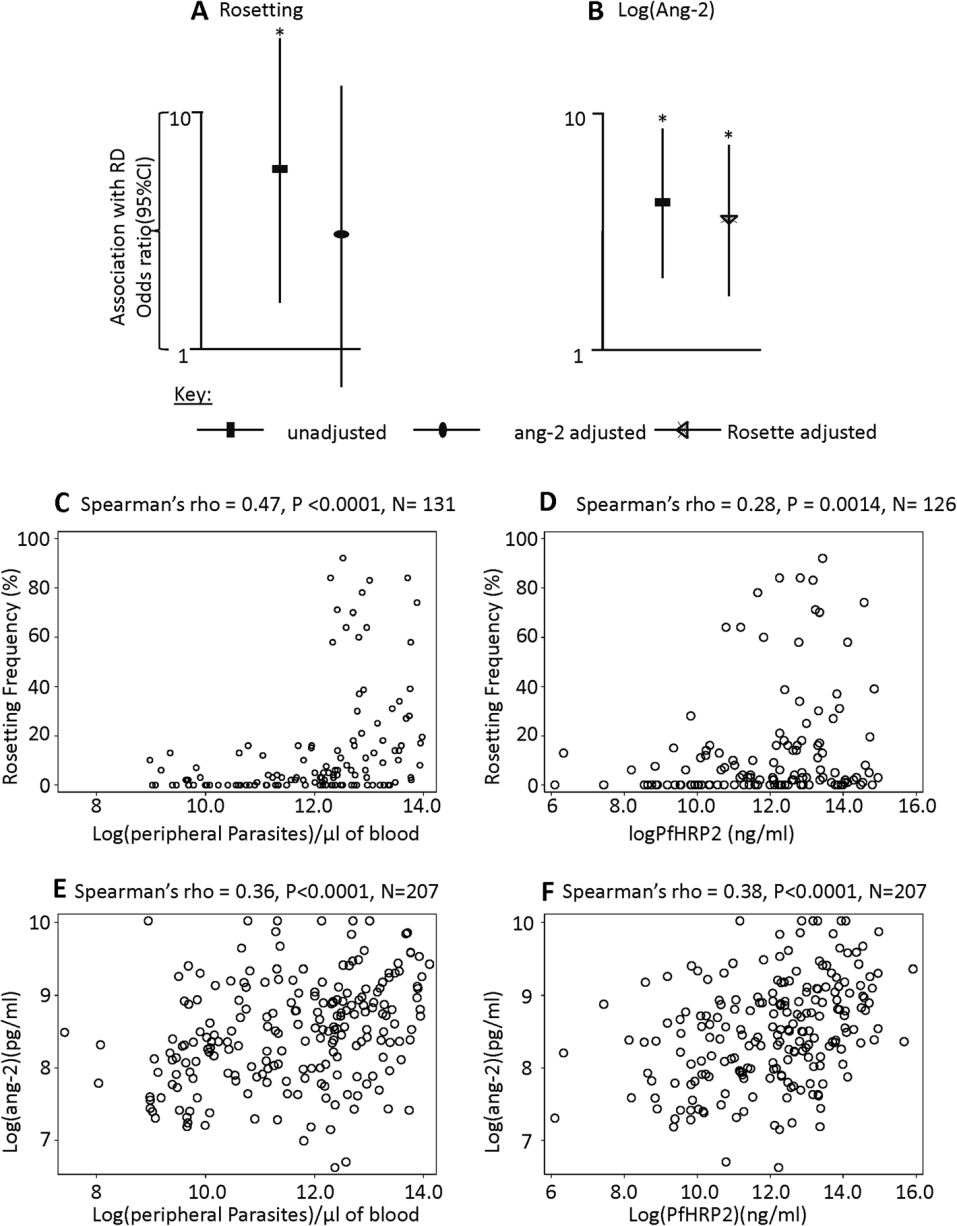
**Relationship between rosette, ang-2 and respiratory distress.** A plot of odds ratio and 95% CI obtained from age-adjusted logistic regression predicting respiratory distress using; **A**) rosetting frequency (unadjusted) and ang-2-adjusted, **B**) ang-2(unadjusted) and rosette frequency-adjusted. Asterisk indicate significance. Scatter plots showing the relationship between **C)** rosetting frequency and peripheral parasitemia, **D)** rosetting frequency and PfHRP2, **E)** ang-2 and peripheral parasitemia, **F)** ang-2 and PfHRP2.

Previously, we found that RD showed evidence for an association with peripheral parasitaemia and rosette frequency within this dataset [10]. Above, we showed ang-2 level is associated with RD (Figure 1B and Table 2), but that appeared not to be a direct relationship between rosetting and ang-2 within the non-severe cases (Figure 2F) suggesting a lack of direct causal relationship. To further explore whether the relationship between rosetting and RD involves endothelial activation measured by plasma ang-2, we used rosette frequency and ang-2 as explanatory variables in age-adjusted logistic regression models predicting RD. We then tested the independence of rosetting frequency and ang-2 in their associations with RD. When ang-2 and rosette frequency were included together in the analysis the OR of the relationship between rosetting and RD dropped by almost a half and the association was no longer statistically significant (Figure 4A and Table 2 model 7). In contrast ang-2 retained its significant associations with RD (Figure 4B, Table 2 model 7). Adding rosetting into a model that considered ang-2 alone showed no evidence of improving the fit (χ^2^ = 2.2, P = 0.14). Taken together these and the results shown in Figure 2F do not support a strong causal link between rosetting and endothelial activation, though Figure 4A is consistent with some level of non-independence. This is potentially well explained by the association between both endothelial activation and rosetting with parasite density (Figure 4C-F) and the non-independent associations of rosetting frequency with RD when adjusted for ang-2 and parasitaemia in age-adjusted logistic regression analysis (Table 2 models 12 and 13).

**Table 2:** Age-adjusted logistic regression models predicting respiratory distress (N = 130)

		**Respiratory distress**	
**Models**	**Explanatory variables**	**OR (95% CI)**	***P***
1 $	Rosette frequency	5.7(1.6, 20.6)	0.008
2 $	Group A-like	1.76(0.49, 6.34)	0.39
3	ang-2	4.1(2.0, 8.55)	0.0001
4 $	parasite density (peripheral)	2.59(1.56, 4.3)	0.0002
5	PfHRP2	1.58(1.18, 2.11)	0.002
6 $	IE surface antibodies	0.98(0.96, 1.0)	0.1
7	Rosette frequency	3.0(0.7, 12.9)	0.14
	ang-2	3.5(1.67, 7.3)	0.001‡
8 $	Rosette frequency	2.0(0.51, 8.3)	0.3
	parasite density (peripheral)	2.4(1.4, 4.0)	0.001‡
9	Rosette frequency	3.54(0.93- 13.42)	0.06
	PfHRP2	1.5(1.11, 2.03)	0.008
10	ang-2	3.2(1.5, 6.99)	0.003‡
	parasite density (peripheral)	2.17(1.30, 3.62)	0.003‡
11	Ang-2	3.35(1.56, 7.20)	0.002‡
	PfHRP2	1.37(1.0, 1.87)	0.044
12	Rosette frequency	1.25(0.26, 6.0)	0.78
	Parasite density (peripheral)	2.1(1.24, 3.6)	0.006‡
	ang-2	3.1(1.4, 6.9)	0.005‡
13	rosette	2.18(0.49, 9.70)	0.31
	PfHRP2	1.34(0.98, 1.83)	0.07
	Ang-2	3.01(1.38, 6.56)	0.005‡

The analysis in this table is similar to that in Table 1 except the outcome of interest is respiratory distress. Ang-2 seems to be in the causal pathway linking rosetting frequency to respiratory distress (models 7, 12 and 13). ‡ = variables that improved the fit of the model using LR χ^2^ improvement test. The analysis in this table is based on 130 samples with rosette frequency data. PfHRP2 data was obtained for 126 out of the 130 samples. $ indicate models published in [10] and repeated here for comparison.

## Discussion

In this study we tested whether a marker of widespread endothelial activation, plasma ang-2 levels throw light on our previously reported associations between expression of group A-like *var genes* and malaria with IC on the one hand and rosetting frequency with RD on the other [10]. Previously, a link between in vivo endothelial dysfunction and severe malaria has been established [21]. In addition, a further study in Malawi revealed that plasma ang-2 is higher in children with retinopathy positive cerebral malaria [25]. As retinopathy is a surrogate marker of cerebral sequestration [5,26,42], this supports the idea that parasite sequestration causes endothelial activation. The recent identification of endothelial protein C receptor (EPCR) as a receptor for severe disease-associated PfEMP1 and the hypothesis that this interaction may drive inflammation [27,28] gives further support to this notion. Following these observations, we explored whether group A-like PfEMP1 may cause higher levels of activation of these cells and release of ang-2, exacerbating inflammation and leading to coma.

Though group A-like *var* subgroup showed a significant but weak association with ang-2 this association dropped out when severe and non-severe cases are considered separately (Figure 2). Moreover, group A-like *var* expression was independently associated with IC when adjusted for ang-2 in a logistic regression model (Figure 3, and Table 1). This is consistent with a model in which 1) IE expressing group A-like PfEMP1 can sequester by binding to ECs in the absence of widespread endothelial activation and inflammation and 2) expression of group A-like *var* contribute to IC in a pathway at least partly independent of widespread ang-2 release.

Recent in vitro studies have shown that IE expressing PfEMP1 subsets containing domain cassette 8 (DC8) and 13 (DC13) can bind to brain endothelial cells (via EPCR) in a manner that is not dependent on the induction of adhesion molecules such as ICAM-1 [15,17,43] that are induced by inflammation. The expression of these PfEMP1 subsets (i.e. DC8 and DC13) in *P. falciparum* isolates sampled from children with severe and non-severe malaria have been shown to be associated with severe malaria [9]. Therefore, in the presence of low host IE surface antibodies, the expression of group A-like PfEMP1 (such as DC13) may give the parasite growth advantage in the initial infection, dominating the sequestered biomass, and causing impaired consciousness before widespread endothelial activation and inflammation occurs.

Our converse observation was that the relationship between ang-2 and IC is not altered by adjustment for group A-like *var* expression (Figure 3 and Table 1). This suggests that parasites expressing group A-like PfEMP1 do not form part of an explanatory link between ang-2 and IC. This in turn suggests that endothelial activation does not confer a growth advantage to parasites expressing group A-like PfEMP-1 through cytoadherence to inducible adhesives molecules such as ICAM-1. ICAM-1 mediated IE cytoadhesion is known to be mediated by a subset of PfEMP1 and has been shown to be associated with cerebral malaria [44-46]. It has been proposed that pathogenesis in this case is mediated through a positive feedback whereby cytoadhesion promotes ICAM-1 expression which further promotes cytoadhesion [47]. If IE adhesion to ICAM-1 or other inducible adhesive molecules is encoded by subsets of both group A and not group A-like PfEMP1 it would provide an explanation for why ang-2 association with IC remains independent of the group A-like *var* expression. We are now in a position to refine approaches to measure *var* gene expression in clinical isolates and explore their associations with different pathogenic mechanisms.

It is important to note that that sequestration per se does not need to play a direct role in endothelial activation for it to be important in disease pathology. Since all IE are thought to sequester, high parasite burden, whatever the underlying cause could lead to endothelial activation independent of the PfEMP1 cytoadhesive profile. This is consistent with the findings of two in vitro studies that used human brain microvascular endothelial cell lines (HBMEC [48,49] and hCMEC/D3 [50] to test whether cytoaherance to ECs is important for endothelial activation. Although they differ in their conclusion on what may be responsible for EC activation, they both agree that binding of IE to the ECs is not necessary for endothelial activation. The study by zougbede et al. further demonstrate that contact between the IE and ECs is not required for endothelial activation [50]. Again in a post-mortem study, Silamut et al. [4] observed, generalised endothelial activation, not confined to sites of parasite sequestration in brain samples from Thai and Vietnamese adults who died from severe malaria, further supporting the possibility of endothelial activation occurring independently of IE binding [4]. Moreover, sera from patients with falciparum malaria were found to induce increased expression of substance P, an observation that was not made with sera from healthy controls [51]. These results raise the possibility that widespread endothelial activation is independent of direct cytoadherence of the IE to the vascular endothelia and that parasite derived circulating factors may be responsible for endothelial activation.

Clearly, parasite density (in the context of falciparum malaria) is likely to be an important determinant of endothelial activation. Parasite density may contribute to endothelial activation by increasing the amount of released microparticles, free haemoglobin, and other molecules. Recently, levels of plasma PfHRP2 (thought to represent parasite load [40,52], though found not useful as a marker of parasite load in a study in Kilifi [53]), was found to differentiate between retinopathy positive CM from retinopathy negative CM [41]. Considering this, the finding that ang-2 is higher in retinopathy positive CM cases compared to retinopathy negative cases [25] is consistent with parasite load being an important determinant of endothelial activation. Moreover, previous work suggests that high parasite burden would lead to an increase in lactic acid production [54], causing acidosis and RD. In this context we found ang-2 to be significantly correlated with parasite density (Figure 4E-F) and base-excess (Additional file 1: Figure S2). Parasite density was shown to be associated with respiratory distress (Table 2) which is a manifestation of metabolic acidosis [30], measured by base excess.

Our *var* gene expression measurements and assessment of rosetting phenotype come from circulating parasites rather than sequestered ones. Since sequestration and microvascular obstruction occur in multiple small areas of the microvasculature and not in a uniform manner [4,55,56], we cannot rule out the possibility of group A-like PfEMP1 involvement in localised EC activation in the brain microvasculature which is not reflected in the measured plasma ang-2 levels. The latter possibility has been suggested by a recently published study that found fibrin deposition occurring more commonly in CM cases than fatal encephalopathic controls that is restricted to microvessels with sequestered IE [27].

## Conclusion

Further studies are needed to determine whether systemic endothelial activation observed in malaria is causally related to cytoadherence or invoked by circulating factors such as microparticles and other IE derived toxins. In Future studies it will be important to explore the role of cytoadhesion to endothelial protein C receptor and use *var* expression assays that more directly measure the levels of domain cassettes known to bind to this receptor.

## Abbreviations

PfEMP1: Plasmodium falciparum erythrocyte membrane protein one; IC: Impaired consciousness; RD: Respiratory distress; SMA: Severe malarial anaemia; IE: Infected erythrocyte; CM: Cerebral malaria; EC: Endothelial cell; HBMEC: Human brain microvascular endothelial cell.

## Competing interests

The authors declare that they have no competing interest.

## Authors’ contributions

PCB, KM: designed the study and provided overall study supervision. GMW, JNM, MO, and PCB: processed samples, performed *var* sequencing, rosetting and IE surface antibody measurements. AA, EK, and MM performed the plasma Ang-2 ELISA: AA, GF and PCB performed the statistical analysis. AA and PCB wrote the manuscript with input from all authors. All authors read and approved the final manuscript.

## Pre-publication history

The pre-publication history for this paper can be accessed here:

http://www.biomedcentral.com/1471-2334/14/170/prepub

## Supplementary Material

Additional file 1: Figure S2 Patients characteristics. **Figure S2.** The relationship between ang‒2 and base‒excess. Click here for file

## References

[B1] Marsh K Forster D Waruiru C Mwangi I Winstanley M Marsh V Newton C Winstanley P Warn P Peshu N Pasvol G Snow R Indicators of life-threatening malaria in African children N Engl J Med 1995 14 21 1399 1404 10.1056/NEJM199505253322102 7723795

[B2] Hanson J Lam SW Mahanta KC Pattnaik R Alam S Mohanty S Hasan MU Hossain A Charunwatthana P Chotivanich K Maude RJ Kingston H Day NP Mishra S White NJ Dondorp AM Relative contributions of macrovascular and microvascular dysfunction to disease severity in falciparum malaria J Infect Dis 2012 14 4 571 579 10.1093/infdis/jis400 22693227

[B3] MacPherson GG Warrell MJ White NJ Looareesuwan S Warrell DA Human cerebral malaria. A quantitative ultrastructural analysis of parasitized erythrocyte sequestration Am J Pathol 1985 14 3 385 401 3893148 PMC1888001

[B4] Silamut K Phu NH Whitty C Turner GD Louwrier K Mai NT Simpson JA Hien TT White NJ A quantitative analysis of the microvascular sequestration of malaria parasites in the human brain Am J Pathol 1999 14 2 395 410 10.1016/S0002-9440(10)65136-X 10433933 PMC1866852

[B5] Taylor TE Fu WJ Carr RA Whitten RO Mueller JS Fosiko NG Lewallen S Liomba NG Molyneux ME Differentiating the pathologies of cerebral malaria by postmortem parasite counts Nat Med 2004 14 2 143 145 10.1038/nm986 14745442

[B6] White VA Malaria in Malawi: inside a research autopsy study of pediatric cerebral malaria Arch Pathol Lab Med 2011 14 2 220 226 21284442 10.5858/135.2.220

[B7] Kyriacou HM Stone GN Challis RJ Raza A Lyke KE Thera MA Kone AK Doumbo OK Plowe CV Rowe JA Differential var gene transcription in Plasmodium falciparum isolates from patients with cerebral malaria compared to hyperparasitaemia Mol Biochem Parasitol 2006 14 2 211 218 10.1016/j.molbiopara.2006.08.005 16996149 PMC2176080

[B8] Warimwe GM Keane TM Fegan G Musyoki JN Newton CR Pain A Berriman M Marsh K Bull PC Plasmodium falciparum var gene expression is modified by host immunity Proc Natl Acad Sci USA 2009 14 51 21801 21806 10.1073/pnas.0907590106 20018734 PMC2792160

[B9] Lavstsen T Turner L Saguti F Magistrado P Rask TS Jespersen JS Wang CW Berger SS Baraka V Marquard AM Seguin-Orlando A Willerslev E Gilbert MT Lusingu J Theander TG Plasmodium falciparum erythrocyte membrane protein 1 domain cassettes 8 and 13 are associated with severe malaria in children Proc Natl Acad Sci USA 2012 14 26 E1791 E1800 10.1073/pnas.1120455109 22619319 PMC3387094

[B10] Warimwe GM Fegan G Musyoki JN Newton CR Opiyo M Githinji G Andisi C Menza F Kitsao B Marsh K Bull PC Prognostic indicators of life-threatening malaria are associated with distinct parasite variant antigen profiles Sci Transl Med 2012 14 129 129ra145 10.1126/scitranslmed.3003247 PMC3491874 22496547

[B11] Bull PC Kortok M Kai O Ndungu F Ross A Lowe BS Newbold CI Marsh K Plasmodium falciparum-infected erythrocytes: agglutination by diverse Kenyan plasma is associated with severe disease and young host age J Infect Dis 2000 14 1 252 259 10.1086/315652 10882604

[B12] Nielsen MA Staalsoe T Kurtzhals JA Goka BQ Dodoo D Alifrangis M Theander TG Akanmori BD Hviid L Plasmodium falciparum variant surface antigen expression varies between isolates causing severe and nonsevere malaria and is modified by acquired immunity J Immunol 2002 14 7 3444 3450 11907103 10.4049/jimmunol.168.7.3444

[B13] Staalsoe T Nielsen MA Vestergaard LS Jensen AT Theander TG Hviid L In vitro selection of Plasmodium falciparum 3D7 for expression of variant surface antigens associated with severe malaria in African children Parasite Immunol 2003 14 8–9 421 427 14651589 10.1111/j.1365-3024.2003.00652.x

[B14] Jensen AT Magistrado P Sharp S Joergensen L Lavstsen T Chiucchiuini A Salanti A Vestergaard LS Lusingu JP Hermsen R Sauerwein R Christensen J Nielsen MA Hviid L Sutherland C Staalsoe T Theander TG Plasmodium falciparum associated with severe childhood malaria preferentially expresses PfEMP1 encoded by group A var genes J Exp Med 2004 14 9 1179 1190 10.1084/jem.20040274 15123742 PMC2211911

[B15] Avril M Tripathi AK Brazier AJ Andisi C Janes JH Soma VL Sullivan DJ Jr Bull PC Stins MF Smith JD A restricted subset of var genes mediates adherence of Plasmodium falciparum-infected erythrocytes to brain endothelial cells Proc Natl Acad Sci USA 2012 14 26 E1782 E1790 10.1073/pnas.1120534109 22619321 PMC3387091

[B16] Buckee CO Recker M Evolution of the multi-domain structures of virulence genes in the human malaria parasite, Plasmodium falciparum PLoS Comput Biol 2012 14 4 e1002451 10.1371/journal.pcbi.1002451 22511852 PMC3325180

[B17] Claessens A Adams Y Ghumra A Lindergard G Buchan CC Andisi C Bull PC Mok S Gupta AP Wang CW Turner L Arman M Raza A Bozdech Z Rowe JA A subset of group A-like var genes encodes the malaria parasite ligands for binding to human brain endothelial cells Proc Natl Acad Sci USA 2012 14 26 E1772 E1781 10.1073/pnas.1120461109 22619330 PMC3387129

[B18] Ponsford MJ Medana IM Prapansilp P Hien TT Lee SJ Dondorp AM Esiri MM Day NP White NJ Turner GD Sequestration and microvascular congestion are associated with coma in human cerebral malaria J Infect Dis 2012 14 4 663 671 10.1093/infdis/jir812 22207648 PMC3266137

[B19] Hollestelle MJ Donkor C Mantey EA Chakravorty SJ Craig A Akoto AO O’Donnell J van Mourik JA Bunn J von Willebrand factor propeptide in malaria: evidence of acute endothelial cell activation Br J Haematol 2006 14 5 562 569 10.1111/j.1365-2141.2006.06067.x 16681646

[B20] Tchinda VH Tadem AD Tako EA Tene G Fogako J Nyonglema P Sama G Zhou A Leke RG Severe malaria in Cameroonian children: correlation between plasma levels of three soluble inducible adhesion molecules and TNF-alpha Acta Trop 2007 14 1 20 28 10.1016/j.actatropica.2007.02.011 17397790

[B21] Yeo TW Lampah DA Gitawati R Tjitra E Kenangalem E Piera K Price RN Duffull SB Celermajer DS Anstey NM Angiopoietin-2 is associated with decreased endothelial nitric oxide and poor clinical outcome in severe falciparum malaria Proc Natl Acad Sci USA 2008 14 44 17097 17102 10.1073/pnas.0805782105 18957536 PMC2575222

[B22] Lovegrove FE Tangpukdee N Opoka RO Lafferty EI Rajwans N Hawkes M Krudsood S Looareesuwan S John CC Liles WC Kain KC Serum angiopoietin-1 and -2 levels discriminate cerebral malaria from uncomplicated malaria and predict clinical outcome in African children PLoS One 2009 14 3 e4912 10.1371/journal.pone.0004912 19300530 PMC2657207

[B23] Amaratunga C Lopera-Mesa TM Brittain NJ Cholera R Arie T Fujioka H Keefer JR Fairhurst RM A role for fetal hemoglobin and maternal immune IgG in infant resistance to Plasmodium falciparum malaria PLoS One 2011 14 4 e14798 10.1371/journal.pone.0014798 21532754 PMC3075246

[B24] Fairhurst RM Bess CD Krause MA Abnormal PfEMP1/knob display on Plasmodium falciparum-infected erythrocytes containing hemoglobin variants: fresh insights into malaria pathogenesis and protection Microbes Infect 2012 14 10 851 862 10.1016/j.micinf.2012.05.006 22634344 PMC3396718

[B25] Conroy AL Glover SJ Hawkes M Erdman LK Seydel KB Taylor TE Molyneux ME Kain KC Angiopoietin-2 levels are associated with retinopathy and predict mortality in Malawian children with cerebral malaria: a retrospective case-control study* Crit Care Med 2012 14 3 952 959 10.1097/CCM.0b013e3182373157 22343839 PMC3284252

[B26] Birbeck GL Beare N Lewallen S Glover SJ Molyneux ME Kaplan PW Taylor TE Identification of malaria retinopathy improves the specificity of the clinical diagnosis of cerebral malaria: findings from a prospective cohort study Am J Trop Med Hyg 2010 14 2 231 234 10.4269/ajtmh.2010.09-0532 20133998 PMC2813163

[B27] Moxon CA Wassmer SC Milner DA Jr Chisala NV Taylor TE Seydel KB Molyneux ME Faragher B Esmon CT Downey C Toh CH Craig AG Heyderman RS Loss of endothelial protein C receptors links coagulation and inflammation to parasite sequestration in cerebral malaria in African children Blood 2013 14 5 842 851 10.1182/blood-2013-03-490219 23741007 PMC3731936

[B28] Turner L Lavstsen T Berger SS Wang CW Petersen JE Avril M Brazier AJ Freeth J Jespersen JS Nielsen MA Magistrado P Lusingu J Smith JD Higgins MK Theander TG Severe malaria is associated with parasite binding to endothelial protein C receptor Nature 2013 14 7455 502 505 10.1038/nature12216 23739325 PMC3870021

[B29] Berkley JA Mwangi I Mellington F Mwarumba S Marsh K Cerebral malaria versus bacterial meningitis in children with impaired consciousness QJM 1999 14 3 151 157 10.1093/qjmed/92.3.151 10326074

[B30] English M Waruiru C Amukoye E Murphy S Crawley J Mwangi I Peshu N Marsh K Deep breathing in children with severe malaria: indicator of metabolic acidosis and poor outcome Am J Trop Med Hyg 1996 14 5 521 524 8940984 10.4269/ajtmh.1996.55.521

[B31] Bull PC Berriman M Kyes S Quail MA Hall N Kortok MM Marsh K Newbold CI Plasmodium falciparum variant surface antigen expression patterns during malaria PLoS Pathog 2005 14 3 e26 10.1371/journal.ppat.0010026 16304608 PMC1287908

[B32] Kirchgatter K Portillo Hdel A Association of severe noncerebral Plasmodium falciparum malaria in Brazil with expressed PfEMP1 DBL1 alpha sequences lacking cysteine residues Mol Med 2002 14 1 16 23 11984002 PMC2039937

[B33] Bull PC Buckee CO Kyes S Kortok MM Thathy V Guyah B Stoute JA Newbold CI Marsh K Plasmodium falciparum antigenic variation. Mapping mosaic var gene sequences onto a network of shared, highly polymorphic sequence blocks Mol Microbiol 2008 14 6 1519 1534 10.1111/j.1365-2958.2008.06248.x 18433451 PMC2440560

[B34] Dustin ML Rothlein R Bhan AK Dinarello CA Springer TA Induction by IL 1 and interferon-gamma: tissue distribution, biochemistry, and function of a natural adherence molecule (ICAM-1) J Immunol 1986 14 1 245 254 3086451

[B35] Weibel ER Palade GE New Cytoplasmic Components in Arterial Endothelia J Cell Biol 1964 14 101 112 10.1083/jcb.23.1.101 14228505 PMC2106503

[B36] Cramer EM Meyer D le Menn R Breton-Gorius J Eccentric localization of von Willebrand factor in an internal structure of platelet alpha-granule resembling that of Weibel-Palade bodies Blood 1985 14 3 710 713 3875375

[B37] Fiedler U Scharpfenecker M Koidl S Hegen A Grunow V Schmidt JM Kriz W Thurston G Augustin HG The Tie-2 ligand angiopoietin-2 is stored in and rapidly released upon stimulation from endothelial cell Weibel-Palade bodies Blood 2004 14 11 4150 4156 10.1182/blood-2003-10-3685 14976056

[B38] Metcalf DJ Nightingale TD Zenner HL Lui-Roberts WW Cutler DF Formation and function of Weibel-Palade bodies J Cell Sci 2008 14 Pt 1 19 27 18096688 10.1242/jcs.03494

[B39] Parikh SM Mammoto T Schultz A Yuan HT Christiani D Karumanchi SA Sukhatme VP Excess circulating angiopoietin-2 may contribute to pulmonary vascular leak in sepsis in humans PLoS Med 2006 14 3 e46 10.1371/journal.pmed.0030046 16417407 PMC1334221

[B40] Dondorp AM Desakorn V Pongtavornpinyo W Sahassananda D Silamut K Chotivanich K Newton PN Pitisuttithum P Smithyman AM White NJ Day NP Estimation of the total parasite biomass in acute falciparum malaria from plasma PfHRP2 PLoS Med 2005 14 8 e204 10.1371/journal.pmed.0020204 16104831 PMC1188247

[B41] Seydel KB Fox LL Glover SJ Reeves MJ Pensulo P Muiruri A Mpakiza A Molyneux ME Taylor TE Plasma concentrations of parasite histidine-rich protein 2 distinguish between retinopathy-positive and retinopathy-negative cerebral malaria in Malawian children J Infect Dis 2012 14 3 309 318 10.1093/infdis/jis371 22634877 PMC3490698

[B42] Beare NA Lewallen S Taylor TE Molyneux ME Redefining cerebral malaria by including malaria retinopathy Future Microbiol 2011 14 3 349 355 10.2217/fmb.11.3 21449844 PMC3139111

[B43] Avril M Brazier AJ Melcher M Sampath S Smith JD DC8 and DC13 var Genes Associated with Severe Malaria Bind Avidly to Diverse Endothelial Cells PLoS Pathog 2013 14 6 e1003430 10.1371/journal.ppat.1003430 23825944 PMC3694856

[B44] Janes JH Wang CP Levin-Edens E Vigan-Womas I Guillotte M Melcher M Mercereau-Puijalon O Smith JD Investigating the host binding signature on the Plasmodium falciparum PfEMP1 protein family PLoS Pathog 2011 14 5 e1002032 10.1371/journal.ppat.1002032 21573138 PMC3088720

[B45] Ochola LB Siddondo BR Ocholla H Nkya S Kimani EN Williams TN Makale JO Liljander A Urban BC Bull PC Szestak T Marsh K Craig AG Specific receptor usage in Plasmodium falciparum cytoadherence is associated with disease outcome PLoS One 2011 14 3 e14741 10.1371/journal.pone.0014741 21390226 PMC3048392

[B46] Bengtsson A Joergensen L Rask TS Olsen RW Andersen MA Turner L Theander TG Hviid L Higgins MK Craig A Brown A Jensen AT A novel domain cassette identifies Plasmodium falciparum PfEMP1 proteins binding ICAM-1 and is a target of cross-reactive, adhesion-inhibitory antibodies J Immunol 2013 14 1 240 249 10.4049/jimmunol.1202578 23209327 PMC3539686

[B47] Berendt AR Simmons DL Tansey J Newbold CI Marsh K Intercellular adhesion molecule-1 is an endothelial cell adhesion receptor for Plasmodium falciparum Nature 1989 14 6237 57 59 10.1038/341057a0 2475784

[B48] Tripathi AK Sha W Shulaev V Stins MF Sullivan DJ Jr Plasmodium falciparum-infected erythrocytes induce NF-kappaB regulated inflammatory pathways in human cerebral endothelium Blood 2009 14 19 4243 4252 10.1182/blood-2009-06-226415 19713460 PMC2925626

[B49] Tripathi AK Sullivan DJ Stins MF Plasmodium falciparum-infected erythrocytes decrease the integrity of human blood-brain barrier endothelial cell monolayers J Infect Dis 2007 14 7 942 950 10.1086/512083 17330783

[B50] Zougbede S Miller F Ravassard P Rebollo A Ciceron L Couraud PO Mazier D Moreno A Metabolic acidosis induced by Plasmodium falciparum intraerythrocytic stages alters blood-brain barrier integrity J Cereb Blood Flow Metab 2011 14 2 514 526 10.1038/jcbfm.2010.121 20683453 PMC3049507

[B51] Chiwakata CB Hort G Hemmer CJ Dietrich M Sera from patients with falciparum malaria induce substance P gene expression in cultured human brain microvascular endothelial cells Infect Immun 1996 14 12 5106 5110 8945553 10.1128/iai.64.12.5106-5110.1996 PMC174495

[B52] Hendriksen IC Mwanga-Amumpaire J von Seidlein L Mtove G White LJ Olaosebikan R Lee SJ Tshefu AK Woodrow C Amos B Karema C Saiwaew S Maitland K Gomes E Pan-Ngum W Gesase S Silamut K Reyburn H Joseph S Chotivanich K Fanello CI Day NP White NJ Dondorp AM Diagnosing Severe Falciparum Malaria in Parasitaemic African Children: A Prospective Evaluation of Plasma PfHRP2 Measurement PLoS Med 2012 14 8 e1001297 10.1371/journal.pmed.1001297 22927801 PMC3424256

[B53] Ochola LB Marsh K Lowe B Gal S Pluschke G Smith T Estimation of the sequestered parasite load in severe malaria patients using both host and parasite markers Parasitology 2005 14 Pt 4 449 458 16174409 10.1017/S0031182005008085

[B54] Day NP Phu NH Mai NT Chau TT Loc PP Chuong LV Sinh DX Holloway P Hien TT White NJ The pathophysiologic and prognostic significance of acidosis in severe adult malaria Crit Care Med 2000 14 6 1833 1840 10.1097/00003246-200006000-00025 10890629

[B55] Dondorp AM Ince C Charunwatthana P Hanson J van Kuijen A Faiz MA Rahman MR Hasan M Bin Yunus E Ghose A Ruangveerayut R Limmathurotsakul D Mathura K White NJ Day NP Direct in vivo assessment of microcirculatory dysfunction in severe falciparum malaria J Infect Dis 2008 14 1 79 84 10.1086/523762 18171289

[B56] Beare NA Harding SP Taylor TE Lewallen S Molyneux ME Perfusion abnormalities in children with cerebral malaria and malarial retinopathy J Infect Dis 2009 14 2 263 271 10.1086/595735 18999956 PMC2757304

