# Supplementary material for: Plasmodium falciparum antigenic variation: relationships between widespread endothelial activation, parasite PfEMP1 expression and severe malaria
Source: BMC Infect Dis. 2014 Mar 28;14:170. doi: 10.1186/1471-2334-14-170 (PMC3986854; doi:10.1186/1471-2334-14-170)
Supplement: Additional file 1: Figure S2 — Patients characteristics. Figure S2. The relationship between ang‒2 and base‒excess. [file 1471-2334-14-170-S1.pdf]

FIGURE S1: Patients characteristics

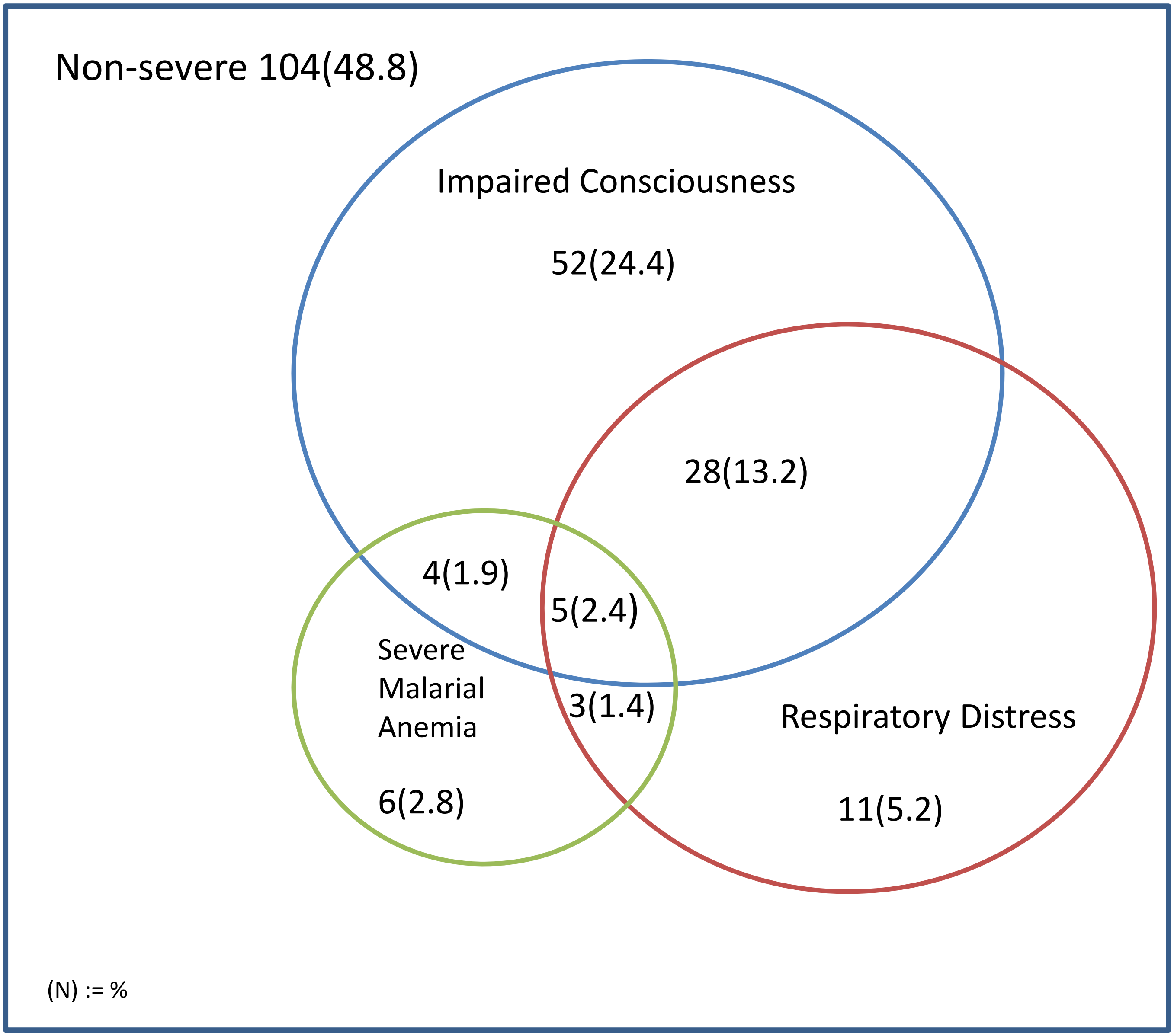

Venn diagram showing the clinical syndromes of the study subjects. IC = 89(41.8) of which CM= 61(28.6), RD=47(22.1) SMA = 18(8.4), and Non-severe=104(48.8)

FIGURE S2:

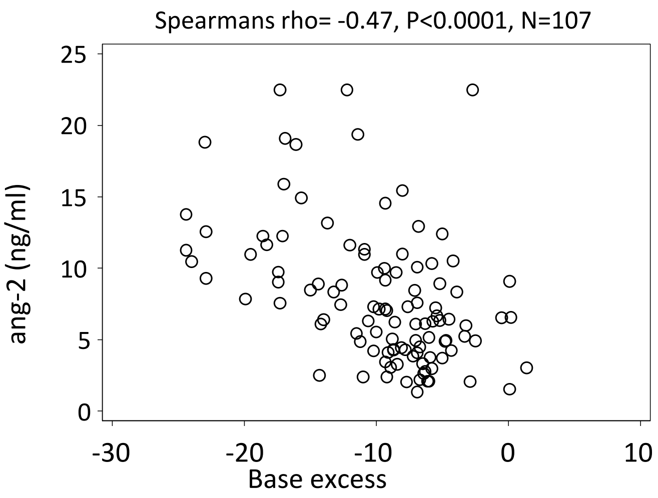

The relationship between ang-2 and base-excess
